# Altered Gene Expression, Mitochondrial Damage and Oxidative Stress: Converging Routes in Motor Neuron Degeneration

**DOI:** 10.1155/2012/908724

**Published:** 2012-05-17

**Authors:** Luisa Rossi, Cristiana Valle, Maria Teresa Carrì

**Affiliations:** ^1^Department of Biology, University of Rome Tor Vergata, Via della Ricerca Scientifica, 00133 Rome, Italy; ^2^Institute for Cell Biology and Neurobiology, CNR, 00100 Rome, Italy; ^3^IRCCS Fondazione Santa Lucia, 00143 Rome, Italy

## Abstract

Motor neuron diseases (MNDs) are a rather heterogeneous group of diseases, with either sporadic or genetic origin or both, all characterized by the progressive degeneration of motor neurons. At the cellular level, MNDs share features such as protein misfolding and aggregation, mitochondrial damage and energy deficit, and excitotoxicity and calcium mishandling. This is particularly well demonstrated in ALS, where both sporadic and familial forms share the same symptoms and pathological phenotype, with a prominent role for mitochondrial damage and resulting oxidative stress. Based on recent data, however, altered control of gene expression seems to be a most relevant, and previously overlooked, player in MNDs. Here we discuss which may be the links that make pathways apparently as different as altered gene expression, mitochondrial damage, and oxidative stress converge to generate a similar motoneuron-toxic phenotype.

## 1. Introduction

Motor neuron diseases (MNDs) are a rather heterogeneous group of diseases, with either sporadic or genetic origin or both, all characterized by the progressive degeneration of motor neurons. All MNDs are primarily axonopathies of the motor neurons in which neuromuscular synapses are early targets of damage and death of motor neurons probably occurs following loss of the neuromuscular junctions [1]. MNDs may manifest as weakness, atrophy of muscles, difficulty in breathing, speaking, and swallowing, with symptoms and severity varying as a consequence of the different involvement of upper or lower motor neurons or both.

The most common and studied form in adults is Amyotrophic Lateral Sclerosis (ALS), followed by Progressive Bulbar Palsy (PBP), the rarer forms being Progressive Muscular Atrophy (PMA) and Primary Lateral Sclerosis (PLS). These conditions seem to form a continuum of diseases since only part of patients have a “pure” phenotype, while others with PBP or PLS eventually develop the widespread symptoms common to ALS [2]. In all these MNDs, onset of symptoms occurs mainly in people aged 40–70. Life expectancy is between 2 to about 5 years after onset in ALS and 6 months to 3 years in PBP, while pure PLS patients may have a normal or near-to-normal life duration. MNDs also include Spinal and Bulbar Muscular Atrophy (SBMA), in which age of onset and severity of manifestations vary from adolescence to old age, but longevity is usually not compromised. Infantile MNDs include Spinal Muscular Atrophy (SMA) with an infantile or juvenile onset and Lethal Congenital Contracture Syndrome (LCCS), causing prenatal death and thus being the most severe form of motor neuron disease.

## 2. Aetiology of MNDs

LCCS1 is an autosomal recessive condition found in communities of the northeastern part of Finland with a prevalence of 1 in 25,250 births [3]. LCCS manifests *in utero* with a marked atrophy of spinal cord motor neurons and fetal immobility due to lack of anterior horn motor neurons, severe atrophy of the ventral spinal cord, and hypoplastic skeletal muscles. It is characterized by total immobility of the fetus, detectable at the 13th week of pregnancy and invariably leading to prenatal death before the 32nd gestational week. The defective gene for LCCS1 is a 16-exon gene coding for GLE1, an mRNA export mediator that is known to interact with the nuclear pore complex and is expressed in the neural tube of 11-day-old mice embryos, specifically in the ventral cell population from which the motor neurons differentiate, and later in other tissues including somites, from which skeletal muscle and bone tissue differentiate [3]. The most frequent mutation in LCCS1 (FinMajor) does not dramatically alter the stability or localization of the protein GLE1 but is predicted to introduce three aminoacid residues in a region that may be critical in the interaction between GLE1 and a motor neuron-specific protein [3].

SMA is the most frequent genetic cause of infant mortality and exists in various forms invariably caused by a genetic defect. Patients with the most common form (proximal SMA) are either deleted for the nine-exon gene *SMN1*, encoding the ubiquitously expressed protein SMN (Survival Motor Neuron) or carry small mutations in the same gene. However, SMA patients always carry at least one copy of the gene SMN2, which encodes the same protein as SMN1 and is only partially functional because of a critical, translationally silent single nucleotide C/T transition inside exon 7 that profoundly affects correct splicing. The clinical severity of SMA ranges from respiratory distress at birth associated with limited life expectancy (SMA1) to onset at older than 10 years and a normal life expectancy (SMA4) and is inversely related to the level of SMN2 compensating for SMN1 deletion [4].

SBMA (also called Kennedy's disease) is an X-linked recessive motor neuron disease in which only lower spinal cord and brain stem motor neurons are affected [5]. SBMA is caused by a polyglutamine expansion in the androgen receptor (AR) [6]; CAG repeat numbers range from 38 to 62 in SBMA patients, whereas healthy individuals have 10 to 36 CAG repeats. Symptoms appear in childhood or early adolescence [7]; SBMA is a rare disease, with the exception of some population in the Vasa region of Western Finland where it was estimated that the prevalence is 13 in 85,000 male inhabitants [8].

While SMA, SBMA, and LCCS1 are invariably familial diseases, adult-onset MNDs are both sporadic and familial. PBP, PMA, and PLS are usually sporadic. ALS occurs sporadically in the majority of cases [9]. Proposed risk factors for ALS include ingestion of high concentrations of *β*-methyl-amino-L-alanine [10], use of cholesterol-lowering drugs [11], intensive physical exercise [12] including football playing [13, 14] and service in the USA Army [15], possibly linked to intermittent occupational hypoxia [16] or to head injury [17–19]. Environmental factors also include cigarette smoking [18, 20], exposure to heavy metals [21], and pesticides or herbicides [22–24]. Approximately 10% of ALS cases is inherited, with multiple autosomal dominant and recessive forms that have been ascribed to mutations in a number of different genes, each of them accounting for a different percentage of cases (Table 1). Interestingly, ALS-associated mutated proteins are implicated in a wide range of cellular processes, from antioxidant response to axonal and vesicular transport, angiogenesis, endoplasmic reticulum (ER) stress and unfolded protein response (UPR), and, most noticeably, to RNA metabolism. 

## 3. Multifactoriality of MNDs: The Role of Altered Gene Expression

At the cellular level, MNDs share features such as protein misfolding and aggregation, mitochondrial damage and energy deficit, excitotoxicity, and calcium mishandling [1], a condition often indicated as multifactoriality. This is particularly well demonstrated in ALS, where both sporadic and familial forms share the same symptoms and pathological phenotype, that are recapitulated in available animal and cell models, with a prominent role for mitochondrial damage and resulting oxidative stress (for an extended Review, see [25]). Oxidative stress is reported also in SMA [26] and reactive oxygen species (ROS) inhibit assembly and activity of SMN complex in a dose-dependent manner [27]. Mitochondrial damage seems to be invariably present in neurodegenerative conditions [28] including SMA [29–32] and SBMA [33], in which mitochondrial dysfunction may be due to the interaction between AR and cytochrome c oxidase subunit Vb (COXVb) [34].

Based on recent data, however, altered control of gene expression seems to be a most relevant, and previously overlooked, player in MNDs.

Several studies addressing epigenetic modifications, transcriptomics, and proteomics of models and tissues from patients indicate that the overall pattern of gene expression is modified in MNDs. Because of the known non-cell autonomous mechanism of death of motor neurons, studies in ALS have been performed in tissues [35, 36] and in neuronal and in nonneuronal cultured cells (astrocytes, muscle) and revealed that most of the deregulated genes are involved in defense responses, cytoskeletal dynamics, protein degradation system, and mitochondrial dysfunction in neurons [37], while the insulin-like growth factor-1 receptor and the RNA-binding protein ROD1 are the most downregulated genes in glia [38]. The pattern is altered also in muscle, in which many of deregulated genes are the same found in surgically denervated muscles, while others appear to be ALS-specific and include proteins clearly involved in the redox response (e.g., metallothionein-2 and thioredoxin-1) [39, 40]. In a recent proteomic study on embryonic stem cell from a severe SMA mouse model differentiated into motor neurons in vitro, Wu et al. reported that 6 proteins are downregulated and 14 upregulated in this model. Most of these proteins belong to the same categories altered in ALS models, that is, are involved in energy metabolism, cell stress response, protein degradation, and cytoskeleton stability [41].

As in other neurodegenerative conditions, alterations of transcription in MNDs may follow altered epigenetic control due to an unbalance between histone acetyl transferases (HATs) and histone deacetylases (HDACs, including sirtuins, SIRTs) activities [42]. These enzymes catalyze forward and reverse reactions of lysine residue acetylation; thus, HATs modify core histone tails thereby enhancing DNA accessibility to transcription factors (TFs), while HDACs activity in general results in transcriptional repression and gene silencing. Interestingly, various TFs, like RelA, E2F, p53, and GATA1, which form part of the transcription initiation complex, are themselves substrates susceptible to the action of HATs and HDACs.

Evidence for the involvement of this kind of regulation in MNDs is accumulating, although still far from definitive, and unspecific HDAC inhibitors such as sodium phenylbutyrate, trichostatin A, and valproic acid have been tested as neuroprotective drugs for the treatment of ALS with some positive result [43–47]. It is interesting to note that valproic acid is also endowed with antioxidative and antiapoptotic properties. However, most likely only selected HDACs participate to onset or propagation of motor neuron damage and thus must be targeted for an effective therapy. This concept is strengthened by the observation that complexes formed by ALS-linked proteins TDP-43 and FUS/TLS control the expression level of HDAC6 [48].

The SMN gene has a reproducible pattern of histone acetylation that is largely conserved among different tissues and species [49] and several HDAC pan-inhibitors such as suberoylanilide hydroxamic acid (SAHA) [50], trichostatin A [51], and the benzamide M344 [52] increase SMN2 transcript and protein levels. Valproic acid is currently tested in phase I and II clinical trials for the treatment of SMA (http://clinicaltrials.gov /). However, valproic acid has also serious adverse effects in cell and mice models for SMA [53, 54] pointing again to the need of inhibition of selected HDACs in MNDs, especially in the light of a recent report that the SMN2 gene is differentially regulated by individual HDAC proteins and silencing of HDAC5 and 6 enhances inclusion of an alternatively spliced exon in SMN2 [55]. Finally, oral administration of the HDAC inhibitor sodium butyrate has been tested also in a transgenic mouse model of SBMA with some positive outcome but only within a narrow range of drug dosage [56].

Epigenetic control of transcription may also occur via methylation by DNA methyltransferases (DNMTs) or histone methyltransferases (HMTs), both using S-adenosylmethionine (SAM o AdoMet) as the methyl donor. DNA methylation in eukaryotes occurs by the covalent modification of cytosine residues (on the fifth carbon) in CpG dinucleotides, leading to gene silencing. Methylation of histones (as well as transcription factors) occurs on lysine or arginine. Methylated lysine residues can carry up to three methyl moieties on their amine group, whereas arginine can be mono- or dimethylated on the guanidinyl group. Lysine methylation of histones is associated with activation or repression of transcription, depending on the degree of methylation and on the residue location [57].

Methylation may be extremely relevant in MNDs if one considers, for instance, that recognition of some Sm proteins by the SMN complex (that mediates the assembly of the Sm proteins onto snRNAs involved in pre-mRNA splicing and histone mRNA processing) is dependent on symmetrical dimethyl arginine modifications of their RG-rich tails [58, 59]. This methylation is achieved by PRMT (protein arginine methyltransferase)5 or by PRMT7, two enzymes that function nonredundantly [60] and utilize SAM as methyl donor. Furthermore, the SMN2 gene is subject to gene silencing by DNA methylation and some HDAC inhibitors including vorinostat and romidepsin are able to bypass SMN2 gene silencing by DNA methylation, while others such as valproic acid and phenylbutyrate are not [61].

Other observations support the concept that MNDs may be considered as “RNA dysmetabolisms” [62]. As reported in Table 1, several of the genetic factors involved in MNDs encode proteins with a role in RNA metabolism, and some overlap may exist among different diseases. For instance, copy number abnormalities of the SMN genes have been reported in sporadic ALS, although decrease of SMN protein in the anterior horn cells of ALS patients may be only a secondary phenomenon [63, 64]. RNA metabolism, however, consists of several intertwined steps, such as pre-mRNA splicing, mRNA transport, translational regulation, or mRNA decay, and the precise RNA pathway that is affected in a single MND remains unknown because virtually every one of the involved RNA-binding proteins has been implicated in more than one of these steps. Thus, it is not clear why motor neurons are so vulnerable to mutations in RNA-binding proteins.

Very recently, familial ALS has been associated with an expansion of a noncoding GGGGCC hexanucleotide repeat in the gene *C9ORF72* [65, 66] that codes for an unknown protein. The transcribed GGGGCC repeat forms intracellular accumulations of RNA fragments in cells in the frontal cortex and the spinal cord from patients carrying the expansion [65]. These RNA *foci* are composed of the expanded nucleotide repeats that may disturb transcription by sequestering RNA-binding proteins involved in transcription regulation as observed for other expanded RNA repeats diseases [67] such as myotonic dystrophy [68]. Interestingly, the GGGGCC sequence also represents a potential binding site of several RNA-binding proteins including hnRNP A2/B1, a TDP-43 interactor [69, 70]. 

## 4. Altered Gene Expression, Mitochondrial Damage, and Oxidative Stress in MNDs: Which Are the Links?

Which are the links among altered gene expression, mitochondrial damage, and oxidative stress in MNDs is not clear yet. While oxidative stress and mitochondrial dysfunction are obviously connected into a vicious cycle in which excess in ROS production may influence the functionality of the organelles, that in turn would produce excess ROS, the connection with altered gene expression in MNDs is still somewhat foggy.

A few considerations may help to shed some light on possible, not mutually exclusive, mechanisms.

In analogy to what has been proposed in development [71] and in cancer [72], an interplay among oxidative stress, thiol redox signaling, and epigenetic modulation by methylation may be critical in motor neurons. The antioxidant capacity of cells is influenced by the production of glutathione (GSH), and increased GSH production influences DNA and histone methylation by limiting the availability of SAM, the cofactor utilized during epigenetic control of gene expression by DNA and histone methyltransferases [71]. The above mentioned forms of methylation, which are relevant in MNDs, are not directly linked, since they involve different enzymes and different targets. However, they all require the same methyl donor, which could be limiting in MNDs.

HDACs themselves seem to be linked to and modulated by oxidative stress. Pan-HDAC inhibition promotes neuronal protection against oxidative stress in a model of glutathione depletion [73], thus suggesting that HDACs are downstream mediator in the mechanisms of toxicity by ROS, while carbonylation of reactive cysteines of some, but not all, class I HDACs causes reduction of histone deacetylase activity and change in histones acetylation and transcription of genes repressed by these HDACs [74]. Thus, oxidative stress may be a modulator of gene expression through the modulation of DNA accessibility.

In turn, the activity of HDACs modulates alternative splicing of human genes when the nascent RNA is still associated with chromatin (in particular the splicing of hundreds of genes is altered upon HDAC inhibition) [75] but also the activity of various TFs. Noticeably, oxidative stress is also a modulator of several TFs and thus ROS and HDACs may concur in the generation of a pathological phenotype through the same mechanism. For instance, as reviewed by Rahman et al. [76], oxidative stress inhibits HDAC activity and activates HAT activity; this leads to NF-*κ*B activation, which, in turn activates proinflammatory mediators. The antioxidant and/or anti-inflammatory effects of thiol molecules (GSH, N-acetyl-L-cysteine and Nacystelyn) and dietary polyphenols (e.g., curcumin and resveratrol) have a role in either the control of NF-*κ*B activation or the modulation of HDAC. Thus, oxidative stress may regulate both TFs and chromatin remodeling which in turn impacts on proinflammatory responses.

Furthermore, SIRTs (class III HDACs) control the expression or the activity of a number of proteins involved in redox regulation (Table 2). Among these proteins, some are mitochondrial and many have been involved in one or more MNDs by transcriptomic/proteomic studies [77–79].

Last, but not the least, we have reported that mitochondrial damage itself is a cause of modification in the abundance of selected splicing variants [80] and that defective RNA metabolism seems to play a role also in SOD1-linked ALS and to descend directly from mitochondrial stress [81].

## 5. A Unifying Mechanism for MNDs?

From what summarized above, it is tempting to speculate that indeed all MNDs are mainly forms of RNA dysmetabolisms. Motor neurons seem to be exceedingly susceptible to defects in RNA transcription or processing; one appealing explanation is that they require that RNA is not only correctly transcribed and spliced, but also correctly transported along axons to neuromuscular junctions (NMJ). While there is no clear demonstration of the presence of mRNAs at the NMJs yet, this process (at least in ALS) might resent from the known alterations in axonal transport that precedes onset of symptoms [102].

However, one form or the other of alteration of RNA expression may have different weight in different MNDs and, most importantly, RNA dysmetabolisms may be a primary event (for instance in SMA or in TDP43- and FUS/TLS-linked ALS) or dysregulation of components of the genetic machinery (the HATs/HDACs system, transcription factors, the splicing complex) may be secondary to oxidative stress or energy failure. In turn, which step is the primary site of damage may dictate the severity of disease (age of onset, progression), and which cell type beside motor neurons is primarily affected may dictate the form of MND. This field surely deserves further investigation aimed to the individuation of novel therapeutic approaches for MNDs.

## Figures and Tables

**Table 1:** Genes involved in MNDs.

Gene	Protein	MND	Main known function
SOD1	Cu, Zn superoxide dismutase	ALS1	Antioxidant enzyme
ALS2	Alsin	ALS2	guanine nucleotide exchange factor for GTPases
SETX	Senataxin	ALS4	DNA/RNA metabolism and repair
SPG11	Spataxin	ALS5	Neuron differentiation and axonal transport
FUS/TLS	Fused in sarcoma	ALS6	RNA binding protein
VAPB	VAMP-associated protein B	ALS8	Trafficking between the endoplasmic reticulum and Golgi apparatus
TDP-43	TAR-DNA-binding protein-43	ALS9	DNA- and RNA-binding protein
ANG	Angiogenin	ALS10	Angiogenesis in response to hypoxia; possibly RNA metabolism
FIG4	PI(3,5)P(2)5-phosphatase	ALS11	Metabolism of phosphatidyl inositol bisphosphate and vesicle dynamic
OPTN	Optineurin	ALS12	Vesicular trafficking
nAChR	Neuronal nicotinic acetylcholine receptor	ALS	Glutamatergic pathway
CHMP2B	Charged multivesicular protein 2B	ALS	Chromatin-modifying protein/charged multivesicular body protein family
VCP	Valosin-containing protein	ALS	Membrane trafficking, organelle biogenesis, maturation of ubiquitin-containing autophagosomes
DAO	D-aminoacid oxidase	ALS	Oxidative deamination of D-aminoacid
UBQLN2	Ubiquilin2	ALS	Ubiquitin-proteasome response
Sig-1R	Sigma-1 receptor	ALS	ER chaperone, modulates calcium signaling through the IP3 receptor
C9ORF72	Unknown	ALS	Unknown
AR	Androgen receptor	SBMA	Androgen receptor
SMN	Survival Motor Neuron	SMA	RNA processing
GLE1	Nucleoporin GLE1	LCCS1	Export of mRNAs containing poly(A)

**Table 2:** Effects of class II HDACs (Sirtuins) on redox-related proteins.

Sirtuin	Target	Effect	Reference
SIRT1 (nucleus and mitochondria)	FOXO3a	↑ Transcriptional activity	[82]
	PGC-1*α*	↑ Transcriptional coactivation	[83, 84]
	HIF1*α*	↓ Transcriptional activity	[85]
	HIF2*α*	↑ Transcriptional activity	[86]
	eNOS	↑ Enzyme activity	[87]
	p53	Mediates transcriptional activity, depending on SIRT1 expression level	[88, 89]
SIRT2 (cytoplasm)	FOXO3a	↑ Transcriptional activity	[90]
SIRT3 (mitochondria)	HIF1*α*	↓ Transcriptional activity	[91]
	SOD2	↑ Enzyme activity	[92, 93]
	OTC	↑ Enzyme activity	[94]
	NDUFA9	↑ Enzyme activity	[95]
	GDH	↑ Enzyme activity	[96]
	IDH2	↑ Enzyme activity	[97]
SIRT4 (mitochondria)	GDH	↓ Enzyme activity	[98]
SIRT5 (mitochondria)	CPS1	↑ Enzyme activity	[99]
SIRT6 (mitochondria)	HIF1*α*	↓ Transcriptional activity	[100]
SIRT7 (nucleoli)	p53	Mediates transcriptional activity, depending on SIRT7 expression level	[101]

CPS1: carbamoyl phosphate synthetase 1; eNOS: endothelial nitric oxide synthase; FOXO3a: Forkhead box O3 a; GDH: glutamate dehydrogenase; HIF1*α*: hypoxia-inducible factor 1, alpha subunit; HIF2*α*: hypoxia-inducible factor 2, alpha subunit; IDH2: isocitrate dehydrogenase 2; NDUFA9: NADH dehydrogenase [ubiquinone] 1 alpha subcomplex subunit 9; OTC: ornithine transcarbamylase; PGC-1*α*: Peroxisome proliferator-activated receptor gamma coactivator 1-alpha; SOD2: superoxide dismutase 2.

## Abbreviations

ALS: Amyotrophic lateral sclerosis
ALS2: Alsin
ANG: Angiogenin
AR: Androgen receptor
CHMP2B: Charged multivesicular protein 2B
COXVb: Cytochrome c oxidase subunit Vb
DAO: D-amino acid oxidase
DNMT: DNA methyltransferase
ER: Endoplasmic reticulum
FIG4: PI(3,5)P(2)5-phosphatase
FUS/TLS: Fused in sarcoma/translocated in liposarcoma
GLE1: Nucleoporin GLE1
GSH: Glutathione
HAT: Histone acetyl transferase
HDAC: Histone deacetylase
HMT: Histone methyltransferase
LCCS: Lethal congenital contracture syndrome
MND: Motor neuron disease
nAChR: Neuronal nicotinic acetylcholine receptor
OPTN: Optineurin;
PBP: Progressive bulbar palsy
PLS: Primary lateral sclerosis
PMA: Progressive muscular atrophy
PRMT5: Protein arginine methyltransferase 5
PRMT7: Protein arginine methyltransferase 7
ROS: Reactive oxygen species
SAHA: Suberoylanilide hydroxamic acid
SAM: S-adenosylmethionine
SBMA: Spinal and bulbar muscular atrophy
SETX: Senataxin
Sig-1R Sigma: 1 receptor
SIRT: Sirtuin
SMA: Spinal muscular atrophy
SMN: Survival motor neuron
SOD1: Cu, Zn superoxide dismutase
SPG11: Spataxin
TDP-43: TAR DNA-binding protein 43
TF: Transcription factor
UBQLN2: Ubiquilin 2
UPR: Unfolded protein response
VAPB: VAMP-associated protein
VCP: Valosin-containing protein.
